# Supplementary material for: C9orf72 polyPR directly binds to various nuclear transport components
Source: eLife. 2024 Mar 14;12:RP89694. doi: 10.7554/eLife.89694 (PMC10939497; doi:10.7554/eLife.89694)
Supplement: Supplementary file 1. [file elife-89694-supp1.docx]

**Supplementary file 1**

**C9orf72 polyPR directly binds to various nuclear transport components**

Hamidreza Jafarinia^1^, Erik Van der Giessen^1^, Patrick R. Onck^1*^

^1^Zernike Institute for Advanced Materials, University of Groningen, Nijenborgh 4, 9747AG Groningen, The Netherlands.

^*^Corresponding author: p.r.onck@rug.nl

Table of Contents

[1 Supplementary tables 2](#_Toc158651700)

# Supplementary tables

**Supplementary file 1a:** Relative hydrophobic strength values of the different amino acids.

| Amino acid | $\varepsilon_{i}$ | Amino acid | $\varepsilon_{i}$ |
| --- | --- | --- | --- |
| A | 0.7 | L | 1 |
| R | 0.005 | K | 0.005 |
| N | 0.33 | M | 0.78 |
| D | 0.005 | F | 1 |
| C | 0.68 | P | 0.65 |
| Q | 0.64 | S | 0.45 |
| E | 0.005 | T | 0.51 |
| G | 0.41 | W | 0.96 |
| H | 0.53 | Y | 0.82 |
| I | 0.98 | V | 0.94 |

**Supplementary file 1b:** Information about the transport components used in this study.

| Transport component | Uniprot ID | Organism | PDB | Seq. length | Res. in the model | Binding partners of each transport component (PDB code) |
| --- | --- | --- | --- | --- | --- | --- |
| Impα1  (KPNA2) | P52292 | Human | 4e4v | 529 | 70-529 | Protein cargoes:  yeast CBP80 cNLS (3uky), Dengue 3 NS5 C-terminal NLS peptide (5fc8), Dengue 2 NS5 C-terminal NLS peptide (5hhg), Zika MR766 NLS (5w41), Nipah virus W protein C-terminus (6bw0), Hendra virus W protein C-terminus (6bw1), XRCC1 NLS peptide (5e6q), Influenza PB2 NLS (4uaf), PARP-2 NLS (5d5k), TPX2 (3knd), Pom121 NLS (4yi0), SART3/TIP110 NLS (5ctt), MAL RPEL (3tpm)  Nup50:  Nup50 (2c1m) |
| Impα3  (KPNA4) | O00629 | Human | 6bvz | 521 | 72-521 | Protein cargoes:  Hendra virus W protein C-terminus crystal forms 1,2,3 (6bw9, 6bwa, 6bwb), Nipah virus W protein C-terminus (6bvv), RCC1(5tbk), Influenza A PB2 NLS (4uae), N-terminal fragment of RanBP3 (5xzx) |
| Impα5  (KPNA1) | P52294 | Human | AlphaFold | 538 | 82-538 | Protein cargoes:  C-terminal domain of Influenza virus PB2 subunit (2jdq)  Nup50:  N-terminus of Nup50 (3tj3) |
| Impα7  (KPNA6) | O60684 | Human | AlphaFold | 536 | 79-536 | Protein cargoes:  Influenza A PB2 NLS (4uad) |
| KAP60 | Q02821 | *S. cerevisiae* | 1bk5 | 541 | 89-541 | Protein cargoes:  SUMO Protease Ulp1p (5h2w), INM protein Heh1 (4xzr), INM protein Heh2 (4pvz), yeast RCC1 (5t94)  Nup2 (yeast Nup50):  Nup2p N-terminal fragment (1un0), Nup2 (2c1t)  Cse1:  CSE1 (1wa5)  RanGTP:  RanGTP (1wa5) |
| CAS | P55060 | Human | AlphaFold | 971 | 1-971 | N.A. |
| Cse1 | P33307 | *S. cerevisiae* | 1z3h | 960 | 1-960 | Impα:  Cse1 (1wa5)  RanGTP:  RanGTP (1wa5) |
| RanGEF  (RCC1) | P18754 | Human | 1a12 | 421 | 1-421 | Ran protein:  Ran (1i2m) |
| RanGAP | P41391 | *S. pombe* | AlphaFold | 386 | 1-386 | RanGTP:  RanGPPNHP (1k5d) |
| NTF2* | P61972  P61970 | Rat  Human | 1oun | 127 | 1-127 | RanGDP:  RanGDP (5bxq, 1a2k) |
| Ran | P62826 | Human | 2mmc | 216 | 1-216 | N.A. |

* The amino acid sequence of NTF2 Rat is identical to the human version.

*NTF2 is a homodimer and the CG model contains 254 residues in total.

**Supplementary file 1c:** Sequences of transport components used for the CG modeling. See also column six of Table S2 for more information about the transport components used in this study.

| NTR name | Amino acid sequence |
| --- | --- |
| Impα1ΔN | NQGTVNWSVDDIVKGINSSNVENQLQATQAARKLLSREKQPPIDNIIRA  LIPKFVSFLGRTDCSPIQFESAWALTNIASGTSEQTKAVVDGGAIPAFI  LLASPHAHISEQAVWALGNIAGDGSVFRDLVIKYGAVDPLLALLAVPDM  SLACGYLRNLTWTLSNLCRNKNPAPPIDAVEQILPTLVRLLHHDDPEVL  DTCWAISYLTDGPNERIGMVVKTGVVPQLVKLLGASELPIVTPALRAIG  IVTGTDEQTQVVIDAGALAVFPSLLTNPKTNIQKEATWTMSNITAGRQD  IQQVVNHGLVPFLVSVLSKADFKTQKEAVWAVTNYTSGGTVEQIVYLVH  GIIEPLMNLLTAKDTKIILVILDAISNIFQAAEKLGETEKLSIMIEECG  LDKIEALQNHENESVYKASLSLIEKYFSVEEEEDQNVVPETTSEGYTFQ  QDGAPGTFNF |
| Impα3ΔN | SLEAIVQNASSDNQGIQLSAVQAARKLLSSDRNPPIDDLIKSGILPILV  CLERDDNPSLQFEAAWALTNIASGTSEQTQAVVQSNAVPLFLRLLHSPH  NVCEQAVWALGNIIGDGPQCRDYVISLGVVKPLLSFISPSIPITFLRNV  WVMVNLCRHKDPPPPMETIQEILPALCVLIHHTDVNILVDTVWALSYLT  AGNEQIQMVIDSGIVPHLVPLLSHQEVKVQTAALRAVGNIVTGTDEQTQ  VLNCDALSHFPALLTHPKEKINKEAVWFLSNITAGNQQQVQAVIDANLV  MIIHLLDKGDFGTQKEAAWAISNLTISGRKDQVAYLIQQNVIPPFCNLL  VKDAQVVQVVLDGLSNILKMAEDEAETIGNLIEECGGLEKIEQLQNHEN  DIYKLAYEIIDQFFSSDDIDEDPSLVPEAIQGGTFGFNSSANVPTEGFQ |
| Impα5ΔN | VITSDMIEMIFSKSPEQQLSATQKFRKLLSKEPNPPIDEVISTPGVVAR  VEFLKRKENCTLQFESAWVLTNIASGNSLQTRIVIQAGAVPIFIELLSS  FEDVQEQAVWALGNIAGDSTMCRDYVLDCNILPPLLQLFSKQNRLTMTR  AVWALSNLCRGKSPPPEFAKVSPCLNVLSWLLFVSDTDVLADACWALSY  SDGPNDKIQAVIDAGVCRRLVELLMHNDYKVVSPALRAVGNIVTGDDIQ  QVILNCSALQSLLHLLSSPKESIKKEACWTISNITAGNRAQIQTVIDAN  FPALISILQTAEFRTRKEAAWAITNATSGGSAEQIKYLVELGCIKPLCD  LTVMDSKIVQVALNGLENILRLGEQEAKRNGTGINPYCALIEEAYGLDK  EFLQSHENQEIYQKAFDLIEHYFGTEDEDSSIAPQVDLNQQQYIFQQCE  PMEGFQL |
| Impα7ΔN | SVITREMVEMLFSDDSDLQLATTQKFRKLLSKEPSPPIDEVINTPRVVD  FVEFLKRNENCTLQFEAAWALTNIASGTSQQTKIVIEAGAVPIFIELLN  DFEDVQEQAVWALGNIAGDSSVCRDYVLNCSILNPLLTLLTKSTRLTMT  NAVWALSNLCRGKNPPPEFAKVSPCLPVLSRLLFSSDSDLLADACWALS  LSDGPNEKIQAVIDSGVCRRLVELLMHNDYKVASPALRAVGNIVTGDDI  TQVILNCSALPCLLHLLSSPKESIRKEACWTISNITAGNRAQIQAVIDA  IFPVLIEILQKAEFRTRKEAAWAITNATSGGTPEQIRYLVSLGCIKPLC  LLTVMDSKIVQVALNGLENILRLGEQEGKRSGSGVNPYCGLIEEAYGLD  IEFLQSHENQEIYQKAFDLIEHYFGVEDDDSSLAPQVDETQQQFIFQQP  APMEGFQL |
| KAP60ΔN | LPQMTQQLNSDDMQEQLSATVKFRQILSREHRPPIDVVIQAGVVPRLVE  MRENQPEMLQLEAAWALTNIASGTSAQTKVVVDADAVPLFIQLLYTGSV  VKEQAIWALGNVAGDSTDYRDYVLQCNAMEPILGLFNSNKPSLIRTATW  LSNLCRGKKPQPDWSVVSQALPTLAKLIYSMDTETLVDACWAISYLSDG  QEAIQAVIDVRIPKRLVELLSHESTLVQTPALRAVGNIVTGNDLQTQVV  NAGVLPALRLLLSSPKENIKKEACWTISNITAGNTEQIQAVIDANLIPP  VKLLEVAEYKTKKEACWAISNASSGGLQRPDIIRYLVSQGCIKPLCDLL  IADNRIIEVTLDALENILKMGEADKEARGLNINENADFIEKAGGMEKIF  CQQNENDKIYEKAYKIIETYFGEEEDAVDETMAPQNAGNTFGFGSNVNQ  FNFN |
| CAS | MELSDANLQTLTEYLKKTLDPDPAIRRPAEKFLESVEGNQNYPLLLLTL  EKSQDNVIKVCASVTFKNYIKRNWRIVEDEPNKICEADRVAIKANIVHL  LSSPEQIQKQLSDAISIIGREDFPQKWPDLLTEMVNRFQSGDFHVINGV  RTAHSLFKRYRHEFKSNELWTEIKLVLDAFALPLTNLFKATIELCSTHA  DASALRILFSSLILISKLFYSLNFQDLPEFFEDNMETWMNNFHTLLTLD  KLLQTDDEEEAGLLELLKSQICDNAALYAQKYDEEFQRYLPRFVTAIWN  LVTTGQEVKYDLLVSNAIQFLASVCERPHYKNLFEDQNTLTSICEKVIV  NMEFRAADEEAFEDNSEEYIRRDLEGSDIDTRRRAACDLVRGLCKFFEG  VTGIFSGYVNSMLQEYAKNPSVNWKHKDAAIYLVTSLASKAQTQKHGIT  ANELVNLTEFFVNHILPDLKSANVNEFPVLKADGIKYIMIFRNQVPKEH  LVSIPLLINHLQAESIVVHTYAAHALERLFTMRGPNNATLFTAAEIAPF  EILLTNLFKALTLPGSSENEYIMKAIMRSFSLLQEAIIPYIPTLITQLT  KLLAVSKNPSKPHFNHYMFEAICLSIRITCKANPAAVVNFEEALFLVFT  ILQNDVQEFIPYVFQVMSLLLETHKNDIPSSYMALFPHLLQPVLWERTG  IPALVRLLQAFLERGSNTIASAAADKIPGLLGVFQKLIASKANDHQGFY  LNSIIEHMPPESVDQYRKQIFILLFQRLQNSKTTKFIKSFLVFINLYCI  YGALALQEIFDGIQPKMFGMVLEKIIIPEIQKVSGNVEKKICAVGITKL  TECPPMMDTEYTKLWTPLLQSLIGLFELPEDDTIPDEEHFIDIEDTPGY  TAFSQLAFAGKKEHDPVGQMVNNPKIHLAQSLHKLSTACPGRVPSMVST  LNAEALQYLQGYLQAASVTLL |
| Cse1 | MSDLETVAKFLAESVIASTAKTSERNLRQLETQDGFGLTLLHVIASTNL  LSTRLAGALFFKNFIKRKWVDENGNHLLPANNVELIKKEIVPLMISLPN  LQVQIGEAISSIADSDFPDRWPTLLSDLASRLSNDDMVTNKGVLTVAHS  FKRWRPLFRSDELFLEIKLVLDVFTAPFLNLLKTVDEQITANENNKASL  ILFDVLLVLIKLYYDFNCQDIPEFFEDNIQVGMGIFHKYLSYSNPLLED  DETEHASVLIKVKSSIQELVQLYTTRYEDVFGPMINEFIQITWNLLTSI  NQPKYDILVSKSLSFLTAVTRIPKYFEIFNNESAMNNITEQIILPNVTL  EEDVELFEDDPIEYIRRDLEGSDTDTRRRACTDFLKELKEKNEVLVTNI  LAHMKGFVDQYMSDPSKNWKFKDLYIYLFTALAINGNITNAGVSSTNNL  NVVDFFTKEIAPDLTSNNIPHIILRVDAIKYIYTFRNQLTKAQLIELMP  LATFLQTDEYVVYTYAAITIEKILTIRESNTSPAFIFHKEDISNSTEIL  KNLIALILKHGSSPEKLAENEFLMRSIFRVLQTSEDSIQPLFPQLLAQF  EIVTIMAKNPSNPRFTHYTFESIGAILNYTQRQNLPLLVDSMMPTFLTV  SEDIQEFIPYVFQIIAFVVEQSATIPESIKPLAQPLLAPNVWELKGNIP  VTRLLKSFIKTDSSIFPDLVPVLGIFQRLIASKAYEVHGFDLLEHIMLL  DMNRLRPYIKQIAVLLLQRLQNSKTERYVKKLTVFFGLISNKLGSDFLI  FIDEVQDGLFQQIWGNFIITTLPTIGNLLDRKIALIGVLNMVINGQFFQ  KYPTLISSTMNSIIETASSQSIANLKNDYVDLDNLEEISTFGSHFSKLV  ISEKPFDPLPEIDVNNGVRLYVAEALNKYNAISGNTFLNTILPQLTQEN  VKLNQLLVGN |
| RanGEF  (RCC1) | MSPKRIAKRRSPPADAIPKSKKVKVSHRSHSTEPGLVLTLGQGDVGQLG  GENVMERKKPALVSIPEDVVQAEAGGMHTVCLSKSGQVYSFGCNDEGAL  RDTSVEGSEMVPGKVELQEKVVQVSAGDSHTAALTDDGRVFLWGSFRDN  GVIGLLEPMKKSMVPVQVQLDVPVVKVASGNDHLVMLTADGDLYTLGCG  QGQLGRVPELFANRGGRQGLERLLVPKCVMLKSRGSRGHVRFQDAFCGA  FTFAISHEGHVYGFGLSNYHQLGTPGTESCFIPQNLTSFKNSTKSWVGF  GGQHHTVCMDSEGKAYSLGRAEYGRLGLGEGAEEKSIPTLISRLPAVSS  ACGASVGYAVTKDGRVFAWGMGTNYQLGTGQDEDAWSPVEMMGKQLENR  VLSVSSGGQHTVLLVKDKEQS |
| RanGAP | MSRFSIEGKSLKLDAITTEDEKSVFAVLLEDDSVKEIVLSGNTIGTEAA  WLSENIASKKDLEIAEFSDIFTGRVKDEIPEALRLLLQALLKCPKLHTV  LSDNAFGPTAQEPLIDFLSKHTPLEHLYLHNNGLGPQAGAKIARALQEL  VNKKAKNAPPLRSIICGRNRLENGSMKEWAKTFQSHRLLHTVKMVQNGI  PEGIEHLLLEGLAYCQELKVLDLQDNTFTHLGSSALAIALKSWPNLREL  LNDCLLSARGAAAVVDAFSKLENIGLQTLRLQYNEIELDAVRTLKTVID  KMPDLLFLELNGNRFSEEDDVVDEIREVFSTRGRGELDELDDMEELTDE  EEDEEEEAESQSPEPETSEEEKEDKELADELSKAHI |
| NTF2 | MGDKPIWEQIGSSFIQHYYQLFDNDRTQLGAIYIDASCLTWEGQQFQGK  AIVEKLSSLPFQKIQHSITAQDHQPTPDSCIISMVVGQLKADEDPIMGF  QMFLLKNINDAWVCTNDMFRLALHNFG |
| Ran | MAAQGEPQVQFKLVLVGDGGTGKTTFVKRHLTGEFEKKYVATLGVEVHP  VFHTNRGPIKFNVWDTAGQEKFGGLRDGYYIQAQCAIIMFDVTSRVTYK  VPNWHRDLVRVCENIPIVLCGNKVDIKDRKVKAKSIVFHRKKNLQYYDI  AKSNYNFEKPFLWLARKLIGDPNLEFVAMPALAPPEVVMDPALAAQYEH  LEVAQTTALPDEDDDL |

#

**Supplementary file 1d:** Parameters for the best linear fits shown in figure 2.

|  | figure 2a ($\boldsymbol{ax+b}$) | | | | figure 2b ($\boldsymbol{ax+b}$) | | | | | |
| --- | --- | --- | --- | --- | --- | --- | --- | --- | --- | --- |
|  | PR20 | | PR50 | | PR20 | | | PR50 | | |
|  | $a$ | $b$ | $a$ | $b$ | $a$ | $b$ | $a$ | | $b$ |  |
| $\boldsymbol{C}_{\mathbf{salt}}\boldsymbol{=200}$ mM | -0.0856 | -0.0027 | -0.0481 | -0.0016 | -0.1216 | -0.0014 | -0.0866 | | -0.0008 |  |
| $\boldsymbol{C}_{\mathbf{salt}}\boldsymbol{=100}$ mM | -0.0955 | -0.0001 | -0.0638 | -0.0002 | -0.0786 | 0.0027 | -0.0754 | | 0.0015 |  |
